# Application of Multivariate Modeling for Radiation Injury Assessment: A Proof of Concept

**DOI:** 10.1155/2014/685286

**Published:** 2014-08-07

**Authors:** David L. Bolduc, Vilmar Villa, David J. Sandgren, G. David Ledney, William F. Blakely, Rolf Bünger

**Affiliations:** Scientific Research Department, Armed Forces Radiobiology Research Institute, Uniformed Services University of the Health Sciences, 8901 Wisconsin Avenue, Bethesda, MD 20889-5603, USA

## Abstract

Multivariate radiation injury estimation algorithms were formulated for estimating severe hematopoietic acute radiation syndrome (H-ARS) injury (i.e., response category three or RC3) in a rhesus monkey total-body irradiation (TBI) model. Classical CBC and serum chemistry blood parameters were examined prior to irradiation (d 0) and on d 7, 10, 14, 21, and 25 after irradiation involving 24 nonhuman primates (NHP) (Macaca mulatta) given 6.5-Gy ^60^Co *Υ*-rays (0.4 Gy min^−1^) TBI. A correlation matrix was formulated with the RC3 severity level designated as the “dependent variable” and independent variables down selected based on their radioresponsiveness and relatively low multicollinearity using stepwise-linear regression analyses. Final candidate independent variables included CBC counts (absolute number of neutrophils, lymphocytes, and platelets) in formulating the “CBC” RC3 estimation algorithm. Additionally, the formulation of a diagnostic CBC and serum chemistry “CBC-SCHEM” RC3 algorithm expanded upon the CBC algorithm model with the addition of hematocrit and the serum enzyme levels of aspartate aminotransferase, creatine kinase, and lactate dehydrogenase. Both algorithms estimated RC3 with over 90% predictive power. Only the CBC-SCHEM RC3 algorithm, however, met the critical three assumptions of linear least squares demonstrating slightly greater precision for radiation injury estimation, but with significantly decreased prediction error indicating increased statistical robustness.

## 1. Introduction

The increasing risks of nuclear and radiological attacks by terrorists as well as the dangers from future industrial and medical radiological accidents emphasize the need for innovative biodosimetry approaches. Large-scale radiation emergencies present a myriad of problems. In mass-casualty scenarios involving radiological-nuclear incidences, it is believed that a significant confounder will be in the taxing of the medical infrastructure due to the sheer number of victims that will likely result. Adding significantly to this burden will be “concerned” individuals but without significant radiation exposure [1]. The identification of radiation biomarkers offers unequivocal potential for performing biodosimetry and formulating medical treatment strategies for specific radiation injuries in both the early hours (h) to days (d) and intermediate 1–4 weeks after the exposure incident [2–5].

Currently, the most practical protocols for estimating hematopoietic acute radiation syndrome (H-ARS) severity from accident victims are those that rely on clinical findings and/or peripheral blood cell counts, such as METREPOL (medical treatment protocols for radiation accident victims) [6]. The METREPOL approach is generally considered the most practical means of assessing radiation injury to guide medical management and categorizes H-ARS into four “response categories” ranging from RC1 (mild) to RC4 (severe) [6–8]. The RITN acute radiation syndrome treatment guidelines [7] incorporate the use of the METREPOL assessment with additional biodosimetry estimators that rely on time-to-vomiting and/or lymphocyte depletion kinetics for estimating ARS [9].

Dose-prediction algorithms have been developed using various biomarkers. For example, an early phase algorithm developed by Goans et al., based on lymphocyte depletion kinetics, was designed for estimating an unknown radiation dose within the first 8 h after receiving an acute whole-body exposure [10]. The algorithm was intended to serve as a first approximation to guide initial medical management. Data used for formulation of the algorithm was obtained from the REAC/TS radiation accident registry, which included 43 gamma exposure cases.

The technique of “multivariate analysis” can be applied to reasonably large datasets [11–14]. State-of-the-art radiation biology and biodosimetry reports have described univariate and bivariate analyses in attempts to correlate the biological effects of radiation doses as prognostic indicators of survival [15, 16]. Ossetrova et al. [15, 16] reported on the application of a “discriminant analysis” technique using blood plasma from a nonhuman primate (NHP) radiation model measured at 1-2 d after radiation exposure. Studies by Blakely and colleagues [17, 18] applied a multivariate “repeated measures” analysis approach, also using data from an NHP radiation model, examining the changes in serum amylase, C-reactive protein (CRP), and hematological blood cell counts measured at 1–4 d after radiation exposure. A recent study by Moroni et al. [19] compared a Gottingen minipig radiation model with radiation data from humans, canines, and baboons for time points ranging between 3 h and 60 d. Changes in C-reactive protein levels and blood recovery profiles were examined. Studies by Meadows et al. [20, 21] demonstrated the utility of using genome-wide expression analysis of peripheral blood (PB) taken at 6 h, 24 h, and 7 d, for generating gene expression profiles in C57BL/6 mice. Meadows et al. showed the potential of PB gene expression profiles for predicting radiation exposure and distinguishing specific doses following TBI. The group also characterized PB signatures of partial-body irradiation exposure using blood drawn at 6 h after irradiation [22] but was unable to predict radiation status based upon the site of the radiation exposure. Baranov and colleagues [23] attempted to improve radiation dose estimation accuracy by developing dose estimation formulas derived from hematological indices from Chernobyl accident patients measured from 4 to 8 d after irradiation exposure. Blood neutrophil, lymphocyte, and platelet kinetics were examined between 0 and 60 d for formulating dose estimation curves based on their nadirs in response to various radiation doses.

A need exists for assessing individuals receiving unknown radiation doses during the intermediate phase (7–21 d). In scenarios in which victims are known to have initially received an unknown radiation dose, early biomarker discrimination is by far the preferred means of assessment [17]. Unfortunately, not all scenarios have involved victims knowledgeable about their initial exposure, such as was the case with an industrial radiation accident in Dakar, Senegal, in 2006 [24]. In these scenarios, the discovery of having been given a radiation dose is sometimes not realized until well after day 7, thus eliminating the opportunity for radiation injury and dose assessment using the classic early phase biomarker panel (CRP, neutrophils, lymphocytes, neutrophil to lymphocyte ratio, and serum amyloid A (SAA)). Intermediate (>1 week after exposure) and long term (months after exposure) biomarkers for dose assessment are therefore necessary.

While these dose assessment approaches have shown utility [15–18, 22, 23], they could be enhanced by an assurance of noncollinearity of the independent variables. Lacking, as well, are weighting methods for the use of several parameters to assess the severity of radiation injury for specific organ or tissue damage. Because of these gaps, potentially effective medical countermeasure techniques are difficult to implement or are not applied appropriately.

Identification of radiation-sensitive biomarkers that are measurable using existing effective analytical techniques would enable medical treatment to be incorporated in a strategic and timely manner [25–28]. The aim of this pilot study was to form a basis for meeting these challenges using a multivariate analytical approach and selection of blood variables that are currently available in the medical diagnostic infrastructure. This paper reports on the proof-of-concept development of algorithms using blood based biomarkers from 7 to 25 d after radiation exposure for estimating a METREPOL H-ARS RC3 condition in a rhesus TBI model. The hypothesis tested was that the application of multivariate analysis can be applied for identifying radiation sensitive complete blood counts (CBCs) and serum blood chemistry parameters in the development of diagnostic H-ARS RC3 algorithms for estimating a METREPOL H-ARS RC3 condition in the time frame between 7 and 25 days after irradiation.

## 2. Materials and Methods

### 2.1. Nonhuman Primates Radiation Model

The NHP radiation model used in this study has previously been described in detail [18, 29]. Research with animals was conducted according to the principles enunciated in the* Guide for the Care and Use of Laboratory Animals *prepared by the Institute of Laboratory Animal Resources, National Research Council. Male rhesus monkeys (*Macaca mulatta*) were housed in individual stainless-steel cages in conventional holding rooms at the Armed Forces Radiobiology Research Institute's (AFRRI) Veterinary Sciences Department in an animal facility accredited by the Association for Assessment and Accreditation of Laboratory Animal Care (AAALAC) International.* Ex vivo *radiation exposures (controls or 0 Gy: *n* = 24; 6.5 Gy TBI ^60^Co *γ* ray at 0.4 Gy min^−1^: *n* = 8) and dosimetry were performed as previously described [18, 29]. All irradiated NHPs received basic clinical supportive care (i.e., oral electrolytes, moist food, etc.). The total body 6.5 Gy radiation dose was considered the equivalent of a METREPOL BM-ARS RC3 condition as outlined in the Medical Management of Radiation Accidents-Manual on the Acute Radiation Syndrome [6–9].

### 2.2. Blood Sampling Analyses

**Figure 1:**
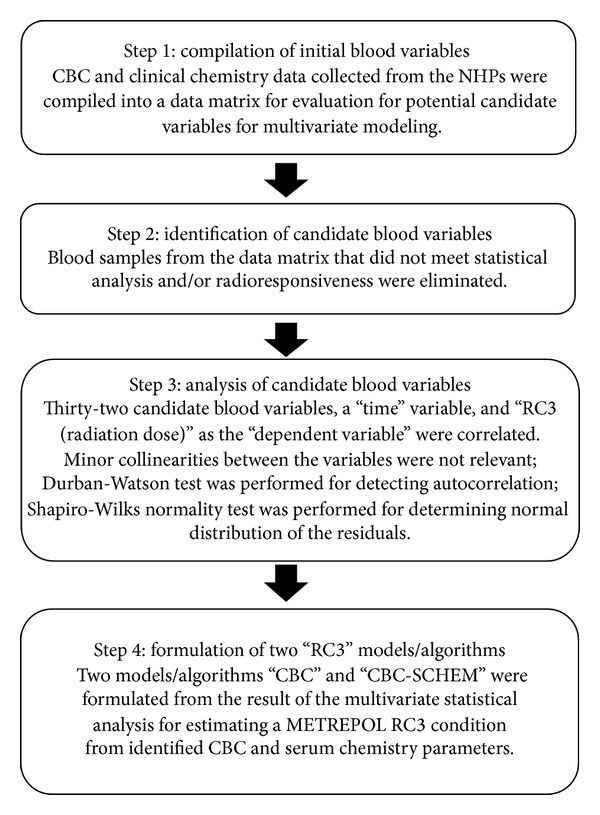
Schematic for formulating a response category 3 (RC3) estimation algorithm. Formulations of the two multivariate models/algorithms were performed in a four-step process: compilation of initial blood variables, identification of candidate blood variables, analysis of candidate blood variables, and the formulation of two “RC3” models/algorithms.

The screening and identification procedures for radiation-responsive candidate blood parameters are outlined in Figure 1.

### 2.3. Compilation of Initial Blood Variables

Blood biosampling (~1.5 mL) for control data was performed twice for all 24 animals over a period of 2 months prior to irradiation. Approximately 1.5 mL of blood was collected from the NHPs that received a 6.5 Gy total body irradiated dose (*n* = 8), on d 7, 10, 14, 17, 21, and 25 after irradiation. The total blood volume draw was less than 10% of the estimated total blood volume based on the animal body weight during the 30-day postirradiation study window. Blood volume draw representing less than 10% over a 1-month period was shown to have negligible influence in NHP ARS studies [30].

A total of 106 permutations of blood parameters consisting of CBCs, serum blood chemistry, and related ratio values were recorded (Table 1). Blood sample parameter values were recorded for the 24 controls NHPs (twice) and 8 of the 24 NHPs irradiated with 6.5 Gy at the 6 postirradiation sampling time points. Sample values were measured and compiled into a data matrix totaling 3,228 data entries. Reference (baseline) concentrations were evaluated for postirradiation sampling time points. Sample values were measured and compiled into a data matrix totaling 3,228 data entries. Reference (baseline) concentrations were evaluated for normality of distribution using MedCalc statistical software (MedCalc Software, Ostend, Belgium). In selected cases, the data were log transformed in order to determine geometric means and 95% confidence limits.

### 2.4. Identification of Candidate Blood Variables

From the data matrix, variables were evaluated for their mean, standard error of the mean (SEM), and standard deviation (SDEV). Variables with SEM values ≤10% of the statistical mean were selected as candidate variables. This procedure was performed in order to imply that the least-squares assumption was met in the fact that random disturbances of each fixed variable of the candidate variables were distributed independently with a mean of zero and common variance (data not shown).

The selected candidate variable datasets were evaluated for their radioresponsiveness determined by a comparison of the irradiated values with the controls using percent differences. Parameters downselected for further multivariate modeling analyses were restricted to only those with differences of ≥10% compared to controls and with SEM of the percent differences of ≤10% (data not shown). Candidate variables that satisfied these criteria were included in the dataset for analysis in a correlation matrix. Conversely, all blood variables that did not meet this criterion were not included in the multivariate analyses. Independent variables that are downselected consisted of 31 blood variables and are presented with an asterisk in Table 1.

### 2.5. Formulation of the “Correlation Matrix” and Analysis of Candidate Blood Variables

A correlation matrix of the 32 prior selected blood parameters along with time and dose was constructed. These 32 variables were then downselected to 9 variables that included the dependent variable (dose) and independent variables of time and 7 of the 32 prior selected blood parameters. The blood parameters were chosen due to their relatively high collinearity with radiation dose as well as their low collinearity with each other to create a more manageable dataset [13]. This dataset was used for modeling radiation injury. The blood candidate variables were tested for correlations with the dependent variable. Pearson correlations were considered between the ranges of 0.25–1.0. Bivariate* r*-squared values were calculated using Statistix 9 analytical software (Statistical Software, Tallahassee, FL) for indicating the predictive power of the independent variables relative to the level of injury from an H-ARS RC3 condition.

### 2.6. Formulation of Two “METREPOL H-ARS RC3 Models”

A multivariate model (with the widely used white blood cell parameters: absolute number of lymphocytes, neutrophils, and platelets as the explanatory variables) was used as the complete blood count “CBC” RC3 model for comparison with a complete blood count serum chemistry “CBC-SCHEM” RC3 model. The CBC-SCHEM model consisted of the three well-established predictors used in the CBC model and four serum chemistry variables. The most efficient combination of the CBC predictors with candidate serum chemistry variables was used to formulate the linear CBC-SCHEM RC3 model for increasing accuracy in estimating a METREPOL RC3 condition.

A “Stepwise Linear Regression” technique (Statistix) was used to determine the best variable combinations for building the CBC-SCHEM model.

### 2.7. Formulation of the CBC RC3 Model

Three commonly employed radiation-sensitive blood variables (biomarkers) were deduced from a literature search; variables with “time” dependency used to formulate a hematology based CBC RC3 model [31–33] included day after radiation dose (TIME), absolute neutrophil count (×10^3^ cells *μ*L^−1^) (ANC), absolute lymphocyte count (×10^3^ cells *μ*L^−1^) (ALC), and absolute platelet count (×10^3^ cells *μ*L^−1^) (APC) [31].

A standard multivariate equation [13, 14] was used as the framework for formulating an RC3 model utilizing the CBC blood variables:

 
*Y* = *α* + (*β*_1_)(*X*_1_)+(*β*_2_)(*X*_2_)+(*β*_3_)(*X*_3_)+(*β*_4_)(*X*_4_) + Residual;
 
*Y* = RC3;
 
*α* = (*α*-coefficient), the *Y* intercept (calculated by Statistix);
 
*β* = (*β*-coefficient), the *β*-coefficient is the amount of change 1 unit of *X* produced in *Y*, which is represented by the slope of the curve (the derived *β*-coefficient was calculated by Statistix for each independent variable used in the model);
 
*X*_1_ = days after radiation dose-variable 1 (TIME);
 
*X*_2_ = CBC-variable 2, neutrophil count (ANC);
 
*X*_3_ = CBC-variable 3, lymphocyte count (ALC);
 
*X*_4_ = CBC-variable 4, platelet count (APC).

### 2.8. Formulation of the CBC-SCHEM RC3 Model

Using the CBC RC3 model as a starting equation, a “CBC-SCHEM” multivariate model was formulated by adding 4 additional independent variables to the CBC RC3 model configuration. The following blood variables were added: relative abundance hematocrit (HCT) in units of percentage and the enzymes aspartate aminotransferase (AST), creatine kinase (CK), and lactate dehydrogenase (LDH) in units per liter.

### 2.9. Statistical Software Application in NHP Radiation Injury Modeling

To construct two multivariate models, mathematical and statistical algorithms from Statistix and Gauss 10 and Gauss *X* (Aptech Systems, Inc., Black Diamond, WA) software were used to compute the coefficients and SEMs of two sets of CBC and blood chemistry variables correlated with a preirradiation (0 Gy) and 6.5 Gy ^60^Co *γ*-radiation dose. Subsequently, the residuals of the two models were compared and examined rigorously for serial errors and autocorrelation (Durbin-Watson statistic (DW)) as well as for constancy of error variance (Shapiro-Wilk (SW) and Breusch-Pagan statistics (BP)). Results from these residual analyses were crucial for determining whether the basic assumptions of linear-least-squares modeling were satisfied by both the CBC and the CBC-SCHEM models. Finally, to determine whether potential problems due to autocorrelation among the independent variables existed, the eigenvalues of the independent variables were computed and evaluated according to criteria developed by Chatterjee and Price [12, 34].

From the multivariate models,* R*-squared values were generated to characterize the independent-variable correlations (relationships) for preirradiation controls (RC0) and the 6.5 Gy radiation dose cohort (RC3). When interpreting an* R*-squared value, it is important to realize that a large value of the* R*-squared or a significant *t*-test statistic does not assure that the data are well fitted [12, 13]. As mentioned above, other tests were performed such as the DW-test for autocorrelation, the SW-test for normality to detect residual patterns, and the BP-test for heteroscedasticity (inconstant error variance). In combination, the results from these three tests provided the rationale for trusting and accepting the calculated SEMs of both the coefficients of the independent variables (the predictor variables) derived parameters such as the predicted values of the dependent variable (radiation dose). These tests provided evidence of no major violations of least-squares-analysis assumptions; hence, secondary evaluations of a single model or any comparisons between models based, for example, on the width of the 95% confidence intervals or the chi square tests were performed.

### 2.10. Formulation of RC3 Algorithms

The CBC and CBC-SCHEM RC3 models were adjusted for estimating the RC3 associated with a 6.5 Gy radiation injury. In this procedure, the “*Y*” variable used in the two-model equations (RC3) was substituted for the calculated “RC3 estimations.”

### 2.11. Deriving the RC3 Estimation Value

The RC3 model served as a template for deriving an RC3 value for the cohort of NHPs given a 6.5 Gy dose. For the RC3 model, the *Y* variable is equal to RC3. The derived RC3 algorithm differs in function from the RC3 estimation model such that *Y* is now equal to an* estimated METREPOL RC value.*

### 2.12. Statistical Testing of the Residuals of the Two RC3 Models

Residuals of the two derived RC3 models (CBC and CBC-SCHEM) were tested for autocorrelation using the DW test for autocorrelation and for significant departure from normality using the SW normality test. Residual profiles also were examined for the two models (to determine systematic residual patterns) using Statistix, as well as the BP-test for heteroscedasticity using Gauss *X*. Statistix was used for calculating eigenvalues for determining the individual noncorrelation score of the independent (predictor) variables used in the models.

Univariate and multivariate receiver operating characteristic (ROC) curve analyses were performed using the ROCCET online tool [35]. The area under the curve (AUC) with 95% confidence limits (CL) was calculated for each blood variable or combination of blood variables using the support vector machine (SVM) approach to show the specificity and sensitivity of biomarker combinations to reflect subgroup differences.

## 3. Results

### 3.1. Selection of Variables for the RC3 Models

Using multivariate analysis, CBC and blood chemistry parameters were evaluated as potential independent variables relative to the effects of a 0 and 6.5 Gy ^60^Co *γ*-radiation TBI dose (RC3). All variables that correlated with the dependent variable were tested against each other for multicollinearity, as shown in Table 2, according to correlation values. The downselection for the variables was based on a high collinearity with radiation and relative low collinearity with each other. The relative order of high correlation (values close to −1 or +1) with radiation was APC > ALC > HCT > ANC > AST > LDH > CK and spanned correlation coefficient values of −0.79 to 0.08. In the case of the selection parameter of low collinearity with each other, the CBC model was limited such that it involved only 3 possible blood count combinations with their correlation coefficients between −0.34 and −0.79. In the case of the CBC-SCHEM model, there are 21 combinations. Each of these selected blood variables when compared with another or all shows two to four combinations with correlation coefficients between >−0.02 and ≤+0.67 with each other.

### 3.2. Radioresponse Time Course for Blood Variables

**Figure 2:**
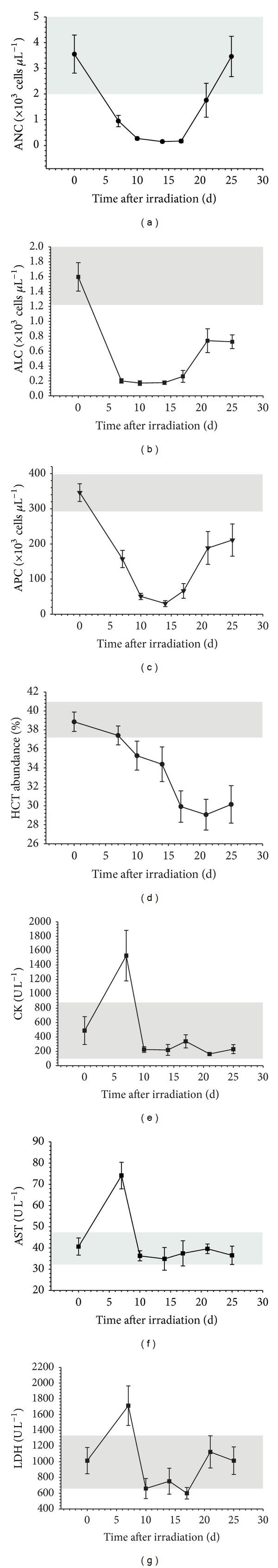
Candidate NHP blood parameters considered in the formulation of the CBC and CBC-SCHEM RC3 models (a) ANC, (b) ALC, (c) APC in (×10^3^ cells *μ*L^−1^), (d) abundance HCT in %, (e) CK, (f) AST, and (g) LDH in UL^−1^. The seven blood parameters were graphed with their standard errors for detecting the radiosensitivity of NHPs to a 6.5 Gy ^60^Co *γ*-radiation dose on d 0 (nonirradiated, *n* = 8) and 7, 10, 14, 17, 21, and 25 d after irradiation (*n* = 8) (shaded areas indicate range between upper and lower 95% confidence levels).

The time course changes for the 7 blood variables used in the models are shown in Figure 2. The main findings shown in Table 3 were that all seven blood variables demonstrated radioresponsiveness at various time points after irradiation. The four CBC variables ANC, APC, ALC, and HCT significantly decreased compared to baseline from day 7 to day 25. The three enzymes AST, CK, and LDH increased compared to baseline on day 7 after irradiation, returning to baseline levels between day 10 and day 25.

### 3.3. Multivariate RC3 Models

Table 4 shows the *α*-coefficients for both the CBC and CBC-SCHEM RIE models determined by stepwise linear regression analysis. The *β*-coefficients were calculated for each independent variable used in the model and are shown in Table 4.

In order to compare the two models' (CBC and CBC-SCHEM) predictive power for radiation injury, the* R*-squared values were determined at 0.91 (91%) (*P* = 0.0001) and 0.93 (93%) (*P* = 0.0001), respectively. Both models explained >90% of the effect a 6.5 Gy ^60^Co *γ*-radiation dose has on the blood variables or the combination of the blood variables with blood chemistry variables.

### 3.4. Testing for Autocorrelation of Variables in the RC3 Models

The fitted sets of the noncollinear independent variables were checked in the two models using the DW test for autocorrelation. Statistical tables revealed that DW test values below 1.5 rejected the hypothesis of the absence of negative autocorrelation. In the range between 1.5 and 1.8, the DW test is considered inconclusive. Both models tested at an inconclusive range between 1.6 and 1.7; that is, there was no definitive evidence for autocorrelation in either model. The SW statistic, however, was more definitive, which indicated a *P* value of 0.03 for the CBC model, clearly rejecting the hypothesis of a normal distribution of the residuals which is a violation of the assumptions of linear-least-squares analyses. In contrast, a *P* value of 0.84 was derived for the CBC-SCHEM model that clearly accepts the hypothesis of normal distribution of the residuals, consistent with the requirements of linear-least-squares analyses.

### 3.5. Testing for Presence of Heteroscedasticity in the RC3 Models

Heteroscedasticity was detected in the CBC model as indicated by the low *P* value of* P*(*W*) = 0.02. Heteroscedasticity was not detected in the CBC-SCHEM model at* P*(*W*) = 0.81. This strengthened the findings from the SW normality test statistic for the CBC-SCHEM model but weakened the SW statistic for the CBC model.

### 3.6. Testing for Multicollinearity in the RC Models

Eigenvalues of the predictor variables were calculated for determining the individual noncorrelation score (collinearity) of the variables used in the models. The sum of the reciprocals of the eigenvalues should not total more than five times the number of predictor variables in the equation. If they do exceed five times, then multicollinearity is of concern [12]. In applying this criterion, the eigenvalues did not suggest significant collinearity in either of the models.

### 3.7. Correlation Analysis and Interpretation

**Figure 3:**
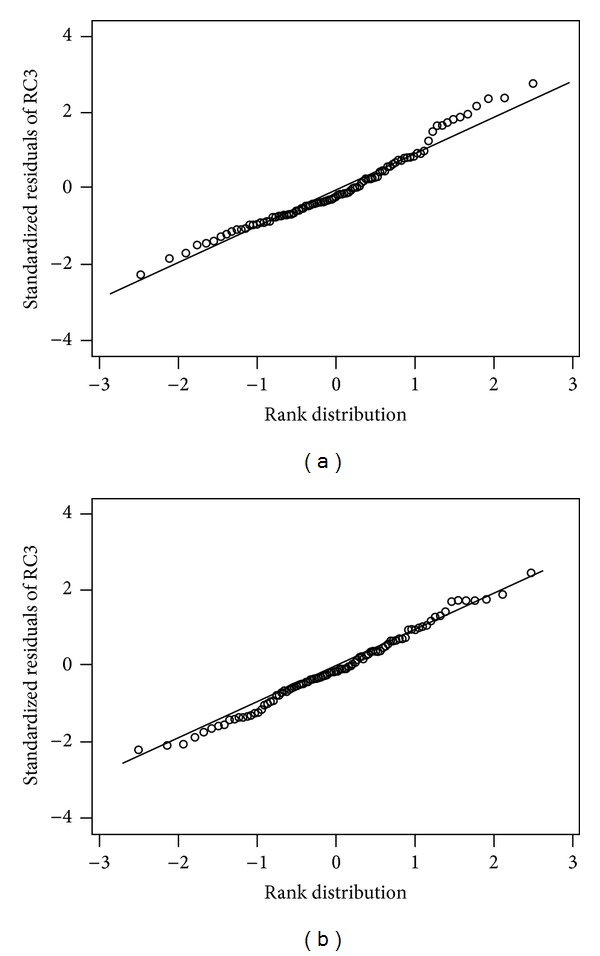
The “CBC”* W* = 0.96 and* P*(*W*) = 0.02 (a) and “CBC-SCHEM”* W* = 0.98 and* P*(*W*) = 0.81 (b): multivariate RC3 models were checked for normal probability and residual patterns. In comparing the residuals of the variables used in the independent variables between the two models, a closer fit to the regression was observed at the tail ends of the CBC-SCHEM RC3 model indicating higher prediction accuracy.

Pearson correlations were performed in order to determine the variables that correlated strongly with the dependent variable yet were noncollinear with each other. Pearson correlation values between independent variables and the dependent variable ranged from −0.34 to 0.67 and −0.58 to 0.87, respectively, in the CBC model, and from −0.25 to 0.77 and −0.79 to 0.26, respectively, in the CBC-SCHEM model (Table 2). As shown in Figure 3, the residuals of the independent variables were closer to the regression in the CBC-SCHEM RC3 model (Figure 3(b)) in comparison with the CBC model (Figure 3(a)) with* W* = 0.96 and* P*(*W*) = 0.02 (hypothesis is rejected of normal distribution of residuals) for the CBC model and* W* = 0.98 and* P*(*W*) = 0.81 (hypothesis is accepted of normal distribution of residuals) for the CBC-SCHEM model.

### 3.8. Interpretation of the ROC Analysis

Table 5 compiles the results of ROC curve analyses for the seven blood variables as potential diagnostic markers for radiation injury. AUC values with 95% CL were calculated at each individual time point for individual biomarkers as well as some combinations, including both the CBC and CBC-SCHEM RC3 models. Between 7 and 17 d after irradiation, ALC, ANC, and APC individually showed great separation of the two doses (AUC ≥ 0.95). At 21 d and 25 d after irradiation of the three, only ALC values maintained the separation (AUC = 0.84 and 1.0, resp.). HCT showed a general increase in AUC between 7 d and 25 d from 0.58 to 0.98, respectively. LDH, CK, and AST showed the highest AUC values at 7 d after irradiation only (AUC ≥ 0.73) and then decreased at 10 d (AUC ≤ 0.57) and remained low through 25 d. The combination of seven biomarkers, the same as used in the CBC RC3 model, showed the highest overall AUC values across all time points.

### 3.9. Testing the RC3 Algorithms

An assessment of the accuracy of the RC3 algorithms (*β*-coefficients) was performed using the same dataset for formulating the RC3 models. Measured blood and time values were entered into the two algorithm templates (shown in Section 2.11).

Calculations related to the estimated RC3 values for either nonradiation (0 Gy) or a 6.5 Gy ^60^Co *γ*-ray TBI dose were then performed using the alpha and beta-coefficients obtained by multivariate analyses from the two RC3 models.

Estimated RC3 assignment accuracies (how close a model estimated an RC3 condition) were compared between the two models. Values for the models were compared by their individual estimated RC3 values and upper and lower 95% confidence and prediction interval bandwidths as shown in Tables 6(a) and 6(b). Both algorithms estimated RC3 spanning 7 to 25 days after irradiation with over 90% predictive power (CBC: 91% ±1.01, *P* = 0.00001, *n* = 92; CBC-SCHEM: 93% ±0.88, *P* = 0.00001, *n* = 92). Only the CBC-SCHEM RC3 algorithm, however, met the critical three assumptions of linear least squares demonstrating slightly greater precision for RC3 estimation, but with significantly increased prediction error (*t* > 108, *P* = 0.00001) suggesting increased robustness of the CBC-SCHEM model.

**Figure 4:**
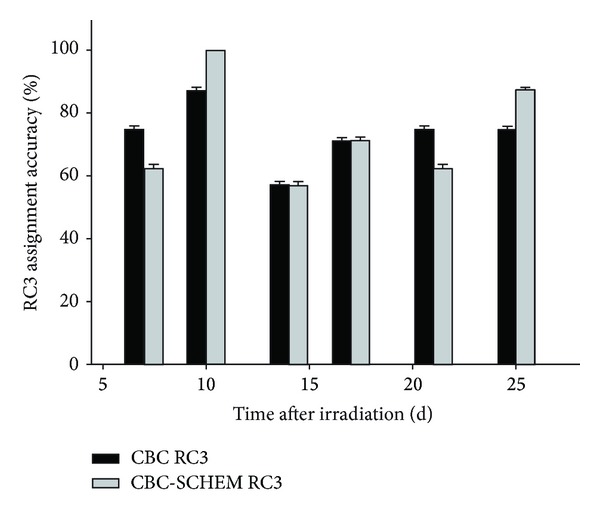
RC3 assignment accuracies derived from the CBC and CBC-SCHEM algorithms were compared with the NHP cohorts (7, 10, 14, 17, 21, and 25 d) after irradiation. Percentages were based on the total number of NHPs that were within the range >2.5–<3.5 for the six postirradiation days (bars represent SD).

Assignment accuracies were derived from the CBC and CBC-SCHEM algorithms and compared with the NHP cohorts at 7, 10, 14, 17, 21, and 25 d after irradiation (Figure 4). The percentages were based on the total number of NHPs that were within the range >2.5–<3.5 for the six postirradiation days. Comparison of the overall assignment accuracies of the two models indicates that neither model is predicted with significantly higher accuracy than the other (CBC overall assignment accuracy = 95.3%, 9 ± 2.58, *n* = 46; CBC-SCHEM overall assignment accuracy = 96.5%, ±2.04, *n* = 46).

When comparing the RC3 assignment accuracies between the CBC and CBC-SCHEM RC3 algorithms, totaling the number of NHPs that were within the ranges of >2.4–<3.5, RC3 assignment accuracy was at 75% and 62.5% for the CBC and CBC-SCHEM, respectively, on day 7. 100% accuracy was reached on day 10 with the CBC-SCHEM algorithm and only 67.5% with the CBC. Both algorithms, however, estimated radiation severity at 57.1% accuracy on day 14 and 71.4% accuracy on day 17. The CBC algorithm estimated better on day 21 at 75% accuracy with the CBC-SCHEM estimating at 62.5%. On day 25, the CBC-SCHEM estimated with greater accuracy at 87.5% while the CBC algorithm estimated radiation severity at only 75% accuracy.

## 4. Discussion

The joint action METREPOL (medical treatment protocols for radiation accident victims) formed within the framework of the Nuclear Fission Safety Program (DG XII Science) of the European Atomic Energy Community was developed to provide guidance for the treatment of radiation accident victims based on experimental and actual data from radiation accident victims. The METREPOL protocols attempt to classify victims suffering from ARS exposure into one of the four response categories (RC), ranging from mild to very severe. The response categorization system is not based on the estimated amount of radiation dose received, but rather on an injury severity score based on a variety of clinical symptoms that are expressed (nausea, vomiting anorexia, fever, headache, blood cell changes, etc.). A flowchart is used as a guide for determining the degree of radiation injury from four specific organs (neurovascular, hematopoietic, cutaneous, and gastrointestinal). Grading codes from 1 to 4 (4 being the most severe) are used for evaluating the severity of radiation injury. The exposed subject is then designated into RC in accordance with the highest grade value [6].

The focus of our study was to develop a multivariate algorithm for calculating the appropriate RC severity for H-ARS with a rhesus monkey TBI model using a 6.5 Gy radiation dose, which based on the literature was predicted to cause RC3 [34]. In place of the METREPOL methodology, time after irradiation and time-dependent blood variable levels would instead be entered into this multivariate algorithm to estimate an H-ARS RC severity.

### 4.1. Multivariate Analysis Application in Estimating RC3 Severity

The main findings in the study were as follows:

1. that classical statistical methods can be applied for developing a rapid simple approach using peripheral blood parameters taken between 7 and 25 days, for estimating a severe H-ARS (i.e., METREPOL RC3),
2. that an RC3 condition can be simulated in an NHP model receiving a total body 6.5 Gy radiation dose,
3. that a proof of concept was demonstrated that a multivariate model composed of seven blood parameters consisting of CBC plus serum chemistry enzymes can estimate RC3 with greater accuracy than a three-parameter CBC model (we thank one of the anonymous reviewers for bringing this very helpful suggestion to our attention).

At present individuals who are judged to have H-ARS RC3 severity would be given cytokine therapy [8], which would be continued daily until neutrophils returned to normal levels typically 3-4 weeks after exposure. The practical application of using multivariate algorithms for predicting RC3 conditions would be in the aid in initiating medical intervention decisions for beginning of cytokine therapy. The CBC-SCHEM model at 10 d was the only model that successfully identified all of the NHPs in the radiation cohort as being correctly assigned to RC3 (Figure 4). Once individuals are categorized as being in RC3 severity, the algorithms can then provide a secondary function to monitor recovery from ARS and treatment efficacy.

The predictive power of how close the models estimated an RC3 (6.5 Gy) radiation dose was evaluated using the Student *t*-test for prediction-confidence intervals. The “confidence interval limit width” mean values confirmed the CBC-SCHEM model as having the highest accuracy. Results from the SW normality test, designed for detecting all departures from normality in the residuals of the fitted equations, were consistent with this conclusion. Typically, the SW normality test rejects the hypothesis of normality in the residuals when a *P* value is less than or equal to 0.05. The CBC model failed this normality test. This allows one to infer with 95% confidence that the fitted equation does not satisfy the requirement for normal distribution of the residuals, thus raising uncertainty about the statistical soundness of the standard deviations of the individual coefficients of any linear regression fit [12]. In comparing “normal probability” between the two models, only the CBC-SCHEM model met the requirement of normal distribution of the residuals.

The ordinary-least-squares (OLS) diagnostic test for heteroscedasticity also was applied to the two regression models for determining whether the variance of the residuals and randomness from the regressions in the two models were dependent on the values of the independent variables. The presence of heteroscedasticity was not detected indicating that all random variables in the sequence had similar variance [12, 35].

The Durbin Watson (DW) test for autocorrelation also was applied to the models for detecting the presence of autocorrelation. Autocorrelation is a systematic (as opposed to random) relationship between residuals separated from each other by a given time lag. The presence of autocorrelation can distort and often understate the SEMs of the alpha- and beta-coefficients (prediction errors) from a regression analysis. The DW-statistic ranges between 0 and 4. A value of 2 indicates no autocorrelation. Values approaching 0 indicate positive autocorrelation and values toward 4 indicate negative autocorrelation. The basic CBC model design generated a DW-test value of 1.61. The expanded CBC-SCHEM model had a slightly higher DW-test value of 1.75. Both these values, however, are in the inconclusive range; that is, there was no definitive evidence of autocorrelation in the residuals in either of the models.

Of the two diagnostic models formulated, the expanded CBC-SCHEM model composed of the seven selected blood variables produced the highest* R*-squared value for estimating radiation injury (93%). The addition of the extra variables AST, CK, HCT, and LDH improved the normal valued* R*-square, which was 2% higher but not statistically significant. However, the prediction limit was slightly improved with no difference in the confidence limit (Table 6) for the CBC-SCHEM algorithm.

The interaction coefficients designated by the beta (*β*) symbol were derived from the correlation software. The beta-coefficients multiply the time and blood variables by how much they are affected by an condition. In the CBC model, the four variables each interact with their specific beta interaction coefficients in estimating radiation injury. The CBC-SCHEM model is composed of eight variables which interact with their specific beta interaction coefficients for deriving its injury estimation. The CBC-SCHEM model has twice the amount (a 100% increase) in the interaction dynamics of variables responding to radiation dose which results in some of the variables no longer counting as highly as they once did in the CBC model.

From the series of statistical tests performed, it was determined that both models are statistically acceptable in terms of* R*-squared, DW-statistic, eigenvalues, and possibly the 95% confidence and prediction intervals. The CBC-SCHEM model showed slightly higher* R*-squared and lower residual sum-of-square (RSS) values and clearly significantly narrower prediction interval limits (decreased prediction error). Based on the variance inflation factor (VIF) statistic and eigenvalues of the predictor's statistic, there is no substantial evidence that the independent variables are collinear. The DW-test did not indicate definitive autocorrelation of residuals or model miss-specification. The error variance was reasonably constant in both models using the OLS heteroscedasticity test but the SW-statistic rejected the hypothesis of equal variances in the basic CBC configuration but not in the expanded CBC-SCHEM model. As expected, the RSS decreased from the CBC model to the CBC-SCHEM model. Therefore, the predictions ± SEMs are more robust and hence reliable and thus more acceptable in the expanded CBC-SCHEM model than in the basic CBC model.

The consequence of having a nonnormal distribution scenario of the residuals around the fitted numbers is that the statistical confidence must be low in the error of predictions. In our case, for the clinical application, the highest level of confidence was desired in these predictions, meaning that the residuals should be higher than* P*(*W*) = 0.05 in the SW test and that the prediction interval widths should be as narrow as possible. In our CBC model, the SW value was at* P*(*W*) = 0.02, indicating a nonnormal distribution; in addition, the prediction interval limits widths were increased relative to the expanded CBC-SCHEM model meaning reduced accuracy in the predictions.

### 4.2. Significance of the 2% Difference in the *R*-Squared Values between the Two Models

In evaluating the residuals and efficacy of the two models, it was concluded that the 2% (±0.88) difference between the two models was not significant in estimating RC3.

### 4.3. Validation from the Receiving Operator Curve Analysis

Validation of the accuracy of the individual variables and the two models was performed using the ROC analysis. The ROC discriminated irradiated (diseased) cases from nonirradiated (normal) cases. The AUC value indicated the degree of separation between irradiated and control values, with 1 indicating a “perfect separation.” The ROC graphically plotted the performance of a binary classifier system with variations occurring throughout its discrimination threshold. The fraction of true positives out of the total actual number of positives was plotted against the fraction of false positives out of the total actual number of negatives [36]. The multiple biomarker receiver operating characteric (M-ROC) analysis validated the inclusion of the additional variables (HCT, LDH, CK, and AST) in the CBC-SCHEM model as improving prediction power (separation). The increase in blood variables from 3 to seven significantly improved the model's separation at the 21- and 25-day time points without causing a loss of compliance with critical least squares assumption.

### 4.4. The Effects of Ionizing Radiation on NHPs

There is currently a large knowledge gap in the effects of ionizing radiation on NHPs. Our study attempted to help fill this gap. Our approach utilized a TBI dose of 6.5 Gy in order to cause RC3 H-ARS severity. This radiation injury, depending on the level of minimal supportive care, is consistent with inducing ~50% mortality was based on the literature [37]. Mortality in a radiation model is dependent not only on several parameters including dose, but also on the level of treatment care and intrinsic radiosensitivity of the individual. We have demonstrated the utility of modeling RC3. This approach was developed using NHP radiosensitive whole blood variables deduced from a standard multivariate analytical model. Our modeling approach demonstrated how standard medical diagnostic information, in this case significant CBC and serum chemistry parameters, could be quantified for estimating a METREPOL H-ARS RC3 condition induced from a 6.5 Gy radiation dose.

Studies modeling biomarkers for characterizing radiation injury in NHPs have been limited. Multivariate discriminant analysis techniques have been applied for estimating a 6.0 Gy radiation exposure in an NHP TBI model using blood plasma collected at 1-2 d after irradiation [16]. The parameters p21 WAF1/CIP 1, Interleukin-6 (IL6), SAA, and CRP were found to be indicators of a 6.0 Gy dose measured at d 1 after irradiation. CRP and SAA were also demonstrated in a similar NHP TBI model as early phase indicators measured at 24 h after irradiation for estimating acute radiation exposures between 1 and 8.5 Gy [15].

A repeated measures approach was applied for estimating a 6.5 Gy dose on an NHP TBI model [17, 18]. CRP, SAA, lymphocytes, and neutrophils to lymphocytes ratio were shown to be indicators of radiation injury between 1 and 15 d after irradiation.

CRP and blood recovery profiles in response to TBI were compared between the Gottingen minipig and NHPs [19]. Changes between early and late phase time points ranging from 3 h to 60 d were compared.

To date, models examining radiation injury on NHPs have primarily focused on the utility of early phase (1–6 d after irradiation) time points for characterizing and predicting TBI injury. A need exists, however, for biomarkers and models for characterizing radiation injury in the intermediate phase (7–25 d). We addressed this challenge by using the practical utility of readily available CBCs and serum chemistry parameters. A multivariate modeling technique was applied using specific noncollinear radiosensitive blood parameters, for estimating an RC3 condition during the intermediate phase. By using combinations of blood parameters that demonstrated low multicollinearity [13, 14] for the development of our RC models, we were able to achieve a high percent accuracy in our characterization of radiation injury (97% ±2) and expand our estimation capability from 7 to 25 d after irradiation. It should be noted that the approach of specifically using noncollinear independent variables for modeling a METREPOL RC has not been reported in the literature.

Our pilot study demonstrated how late phase (>7 d) hematology and serum chemistry biomarkers could be used in unison for estimating a METREPOL H-ARS RC3 condition. Moreover, the integration of molecular biomarkers that are known to manifest in the prodromal and/or late ARS phases (Flt3 ligand, citrulline, C-reactive protein, and serum amylase IL-6) may contribute to our algorithm design in improving accuracy in determining the degree of an RC condition at various time points [15, 28, 38–40].

An algorithm that was sensitive enough to detect the prodromal symptoms of a response category suggests the possibility of initiating early treatment. For example, if the early symptoms of RC4 could be detected in time, appropriate treatment could then be promptly initiated such as in administering blood cells transfusions to bridge the accident victim until bone marrow transplant therapy can reconstitute bone marrow stem cells.

### 4.5. Limitations and Alternatives

The archival data used in the present study originated from a previous experiment performed at AFRRI using NHPs exposed to a single total body 6.5 Gy radiation dose sufficient to cause severe H-ARS. The study design was focused specifically on determining survival outcome of NHPs after radiation exposure. All of the NHPs survived, which was likely due to the excellent postirradiation basic clinical supportive care.

The AFRRI experimental study protocol was not ideal for generating data that could later be utilized for modeling changes in radiation injury. Because of the limited 6.5 Gy cohort dataset, it was only possible to design an algorithm for estimating a METREPOL H-ARS RC3 condition. Ideally, it would have been better to have had a greater number than 8 NHPs and to have designed our algorithm from a systemic gradient of radiation doses for potentially robust estimations of all the response METREPOL categories.

The number of postirradiation days available for blood sampling was also a limiting factor. This limitation compromised the possibility of identifying all possible sensitive hematology subsets associated with an RC3 condition from a 6.5 Gy TBI dose. Ideally, earlier (before 7 d after irradiation) and later time points (after d 25) would have permitted expanded early and late phase estimations of the RC3 profile. This approach would have demonstrated greater relevance for rapid and more reliable medical assessments.

The selection criterion for candidate variables to formulate the CBC-SCHEM RC3 model also may have been a limiting factor in the fact that it may have been too stringent and thus eliminated other significant and potentially highly predictive variables. In the study criterion, only radiosensitive parameters with SEM values ≤ 10% of the statistical mean were considered for modeling. Importantly, the lack of an independent dataset (not used in the modeling efforts) to fully test the efficiency and accuracy of the radiation injury estimation algorithm also was a limitation. Because of the absence of an additional blood component dataset, we were limited to the existing dataset for testing the precision of the derived algorithms.

Despite the considerable limitations of this study, however, we demonstrated that our original hypothesis was correct such that the application of multivariate analysis can be applied for identifying radiation sensitive complete blood counts (CBCs) and serum blood chemistry parameters in the development of diagnostic H-ARS RC3 algorithms for estimating a METREPOL H-ARS RC3 condition in the time frame between 7 and 25 days after irradiation.

This pilot study demonstrated the potential utility and power of the multivariate modeling approach for diagnosing an RC3 condition based on simple whole blood cell and biochemical parameters. The development of predictive algorithms based on multivariate modeling offers considerable biodosimetry applications. The modeling and estimation techniques reported in this paper can be applied to both linear and nonlinear models based on raw data from any mammalian cellular, biochemical, and molecular parameters.

## 5. Summary

Taken together, the results from graphing of the RC3 assignment accuracies demonstrate the utility of using a multivariate approach for developing RC3 estimation algorithms for utility between 7 and 25 days.

From our study we have shown that some blood variables are more radiation sensitive than others and that certain combinations of variables will work better for estimating an RC3 condition than others. It is likely that some variables may not demonstrate sensitivity at lower radiation doses, while others will. Variables with sensitivity to relatively low radiation doses, however, may demonstrate some degree of overlap with the higher doses, which would render the use of these variables impractical for modeling. This has yet to be determined. We believe the next logical step would be to model a full dose gradient for determining the optimal combination of variables for detecting the three additional METREPOL response categories.

From a cost effectiveness standpoint, at present, variables from both the CBC and serum chemistry panels are needed for building a statistically sound model. However, after modeling data from a gradient of radiation doses, new combinations of variables may be discovered. It may be possible to develop an accurate H-ARS RC algorithm from strictly hematology parameters, in which case the modeling procedure would be not only simpler and faster but also more cost effective.

## Figures and Tables

**Table 1:** The 106 CBC, blood chemistry parameters, and related ratios based from the 7 time points (0, 7, 10, 14, 17, 21, and 25 d after irradiation). Variables marked with an asterisk indicate the 32 selected for entry in the correlation matrix.

CBC panel parameters	Blood chemistry panel parameters	Ratios of CBC and blood chemistry parameters	
∗Hematocrit (relative volume of erythrocytes) (HCT)	∗Alanine transaminase level (ALT)	TRIGL/TP	CK/ALB
∗Hemoglobin concentration (HGB)	Albumin level (ALB)	#BASO/WBC	CK/ALB
∗Mean corpuscular (erythrocyte) volume (MCV)	∗Alkaline phosphatase level (ALKP)	#EOS/WBC	CK/TP
∗Mean corpuscular hemoglobin (MCH)	Amylase level (AMYL)	#LUC/WBC	CK/TP
∗Mean corpuscular hemoglobin concentration (MCHC)	∗Aspartate aminotransferase level (AST)	ALC/WBC	CO2/PO4
∗Mean platelet (thrombocyte) volume (MPV)	Bilirubin level (BILI)	#MONO/WBC	GGT/ALB
∗Number of basophils (# BASO)	∗Blood urea nitrogen level (BUN)	#RETIC/WBC,	GGT/TP
∗Number of eosinophils (# EOS)	Calcium level (Ca)	%EOS/WBC	GLU/ALB
∗Number of leucocytes (# LUC)	∗Carbon dioxide concentration (CO2)	%LYMPH/WBC	HCT/ALB
∗Number of lymphocytes (# ALC)	Chloride level (Cl)	%NEUT/WBC	HGB/RBC
∗Number of mononuclear cells (# MONO)	Cholesterol Level (CHOL)	%RETIC/WBC	K/Na
∗Number of neutrophils (# ANC)	∗Creatine kinase level (CK)	ALB/TP	LDH/ALB
∗Number of reticulocytes (# RETIC)	∗Creatinine level (CR)	ALKP/ALB	LDH/TP
∗Percentage of basophils (% BASO)	Gamma-glutamyl transferase level (GGT)	ALKP/TP	LPS/ALB
∗Percentage of eosinophils (% EOS)	Glucose level (GLU)	ALT/ALB	MCH/RBC
∗Percentage of leukocytes (% LUC)	∗Lactate dehydrogenase level (LDH)	ALT/TP	MCHC/RBC
∗Percentage of lymphocytes (ALC)	Lipase level (LPS)	AMYL/ALB	MCV/WBC
Percentage of mononuclear cells (% MONO)	Phosphate level (PO4)	AST/ALB	MPV/WBC
∗Percentage of neutrophils (% NEUT)	∗Potassium level (K)	AST/TP	Na/K
∗Percentage of reticulocytes (% RETIC)	Sodium level (Na)	BASO/%LUC	ANC//WBC
∗Platelet count (# APC)	Total protein level (TP)	BILI/ALB	APC/WBC
∗Red blood cell count (# RBC)	Triglyceride level (TRIGL)	BILI/TP	PO4/CO2
∗White blood cell count (# WBC)	∗Uric acid level (URIC)	BUN/ALB	RBC/WBC
		Ca/ALB	TP/ALB
		Ca/PO4	TRIG/ALB
		Ca/TP	TRIGL/CHOL
		CHOL/ALB	URIC/ALB
		CHOL/TP	URIC/BUN
		CHOL/TRIG	URIC/TP
		Cl/Na	WBC/RBC

**Table 2:** Multivariate correlation values for the “CBC” (bold) and the “CBC-SCHEM” (italic) RC3 models.

Correlation coefficients								
Parameters	Rad dose	Time	ANC	ALC	APC	AST	CK	HCT
Rad dose		**0.87**	**−0.58**	**−0.77**	**−0.79**			
Time	*0.87 *		**−0.34**	**−0.58**	**−0.60**			
ANC	*−0.57 *	*−0.33 *		**0.60**	**0.66**			
ALC	*−0.77 *	*−0.59 *	*0.61 *		**0.67**			
APC	*−0.79 *	*0.04 *	*0.66 *	*0.67 *				
AST	*0.26 *	*−0.13 *	*−0.10 *	*−0.26 *	*−0.12 *			
CK	*0.08 *	*−0.77 *	*−0.04 *	*0.16 *	*−0.03 *	*0.60 *		
HCT	*−0.68 *	*0.06 *	*0.31 *	*0.54 *	*0.57 *	*−0.02 *	*0.07 *	
LDH	*0.14 *	*0.06 *	*0.12 *	*−0.12 *	*0.06 *	*0.77 *	*0.58 *	*0.04 *

**Table 3:** Quantitative measures to assess radioresponses for selected seven blood variables including: a) initial time of change and relative fold change, b) nadir time window and relative nadir fold changes, and c) time until return to baseline levels.

Blood parameters	Baseline range	Initial time of change (day)	Fold change					Nadir time (day)	Nadir values fold change					Return to baseline time (day)
				RSD ∗(+/−)	*t*	*P*	*n*			RSD ∗(+/−)	*t*	*P*	*n*	
ANC	2.82–4.30 × 10^3^ *μ*L^−1^	7	3.7	1.6	−3.25	0.01	8	10–17	23.89	8.83	−4.3	0.004	8	21
ALC	1.41–1.78 × 10^3^ *μ*L^ −1^	7	3	0.09	−8.35	0.01		7–17	7.98	1.53	−8.5	0.0001	8	25
APC	321.82–370.74 × 10^3^ *μ*L^ −1^	7	2.19	0.37	−5.32	0.0001	8	10–17	11.32	3.26	−13	0.0001	8	—
HCT	37.93–39.99 (%)	10	1	4.54	−1.65	0.01		17–25	1.33	0.08	−5.9	0.0006	8	—
AST	36.86–44.78 U/L^−1^	7	1.82	0.23	3.81	0.006	8	—	—	—	—	—	8	10
CK	304.57–681.71 U/L^−1^	7	3.12	1.4	1.65	0.04	8	—	—	—	—	—	8	10
LDH	846.41–1185.83 U/L^−1^	7	1.68	0.37	2.34	0.05	8	—	—	—	—	—	8	10

*Relative Standard Deviation (RSD).

**Table:** (a) “CBC” RC3 model

RC3 = 1.93 + (0.09) (TIME) + (−0.06) (ANC) + (−0.36) (ALC) + (−2.685 × 10^−3^) (APC)			
*R* ^ 2^ = 0.908, *P* = 0.00001, *n* = 92, *F* = 39.3, SE of the estimate = ±1.01			

Predictor	Value	*t* value	*P* value

*α*	1.93	12.61	0.00
TIME *β*1	0.09	13.06	0.00
ANC *β*2	−0.06	−1.87	0.06
ALC *β*3	−0.36	−4.53	0.00
APC *β*4	−2.685 × 10^−3^	−4.81	0.00

**Table:** (b) “CBC-SCHEM” RC3 model

∗RC3 = 0.42 + (0.11) (TIME) + (−0.06) (ANC) + (−0.26) (ALC) + (−2.787 × 10^−3^) (APC) + (0.01) (AST) + (1.968 × 10^−5^) (CK) + (0.02) (HCT) + (−8.682 × 10^−5^) (LDH)			
*R* ^ 2^ = 0.933, *P* = 0.00001, *n* = 92, *F* =36, SE of the estimate = ±0.88			

Predictor	Value	*t* value	*P* value

*α*	0.42	0.85	0.39
TIME *β*1	0.11	12.82	0.00
ANC *β*2	−0.06	−2.06	0.04
ALC *β*3	−0.26	−3.50	0.00
APC *β*4	−2.787 × 10^−3^	−5.52	0.00
AST *β*5	0.01	2.71	0.00
CK *β*6	1.968 × 10^−5^	1.81	0.07
HCT *β*7	0.02	1.79	0.07
LDH *β*8	−8.682 × 10^−5^	−0.61	0.54

*Note.* The *t* value represents the ratio of the *β*-coefficient over its SE. The *P* value represents the significance of the *t* value.

*(Adding of CK and LDH enables the model to pass the requirements of linear-least-squares analysis.)

See text 2.7 for units values of the variables shown in both algorithms.

**Table 5:** Receiving operator curve analysis of single and combination of blood variables at the six time points 7, 10, 14, 17, 21, and 25 d after irradiation equations.

ROC AUC values at 95% CL, comparison of RC0 and RC3								
Time after irradiation, d								
Blood variable combination		7 d	10 d	14 d	17 d	21 d	25 d	Pooled
ALC	**AUC**	**1**	**1**	**1**	**1**	**0.85**	**1.0**	**0.98**
	95% CL					0.04–1.00	0.93–1.00	0.94–1.00
ANC	**AUC**	**0.96**	**1**	**1**	**1**	**0.43**	**0.56**	**0.88**
	95% CL	0.90–1.00				0.00–0.90	0.20–0.83	0.77–0.98
APC	**AUC**	**0.99**	**1**	**1**	**1**	**0.76**	**0.67**	**0.94**
	95% CL	0.97–1.00				0.00–1.00	0.00–1.00	0.85–1.00
HCT	**AUC**	**0.58**	**0.74**	**0.88**	**0.99**	**0.99**	**0.98**	**0.92**
	95% CL	0.05–1.00	0.02–0.98	0.08–0.99	0.98–1.00	0.98–1.00	0.94–1.00	0.84–0.99
LDH	**AUC**	**0.74**	**0.51**	**0.54**	**0.48**	**0.45**	**0.47**	**0.42**
	95% CL	0.04–0.97	0.25–0.78	0.24–0.84	0.17–0.85	0.16–0.86	0.20–0.77	0.29–0.66
CK	**AUC**	**0.91**	**0.49**	**0.48**	**0.51**	**0.5**	**0.47**	**0.50**
	95% CL	0.02–0.98	0.34–0.65	0.17–0.85	0.12–0.86	0.30–0.71	0.24–0.76	0.36–0.65
AST	**AUC**	**0.96**	**0.50**	**0.58**	**0.56**	**0.50**	**0.54**	**0.44**
	95% CL	0.92–1.00	0.30–0.75	0.20–0.82	0.09–0.91	0.19–0.81	0.33–0.75	0.20–0.65

“CBC” RC3 model								
ALC, ANC, APC	AUC	1	1	1	1	0.87	0.92	0.97
	95% CL					0.51–1.00	0.61–1.00	0.91–1.00

“CBC-SCHEM” RC3 model								
ALC, ANC, APC, HCT, LDH, CK, AST	**AUC**	**1**	**1**	**1**	**1**	**0.95**	**0.96**	**0.99**
	95% CL	0.99-1.00	0.95–1.00	0.95–1.00	0.98–1.00	0.76–1.00	0.82–1.00	0.97–1.00

**Table 6:** RC3 estimations.

CBC∗				CBC-SCHEM^†^			
Day	Estimated Response Category	95% Prediction Limit Width	95% Confidence Interval Limit Width	Day	Estimated Response Category	95%Prediction Limit Width	95%Confidence Interval Limit Width
7	**1.73**	1.90	0.39	7	**2.26**	1.76	0.63
	**2.56**	1.89	0.32		**2.54**	1.70	0.39
	**2.93**	1.90	0.30		**2.72**	1.88	0.33
	**2.78**	1.90	0.60		**2.85**	1.67	0.54
	**3.17**	1.89	0.63		**3.20**	1.68	0.57
	**2.09**	1.88	0.33		**2.48**	1.99	0.44
	**2.57**	1.89	0.33		**2.34**	1.68	0.39
	**3.16**	1.93	0.37		**3.00**	1.84	0.42

10	**3.33**	1.89	0.35	10	**3.16**	1.68	0.41
	**3.31**	1.89	0.39		**3.22**	1.68	0.36
	**3.50**	1.90	0.53		**3.48**	1.68	0.54
	**2.24**	1.90	0.35		**3.08**	1.68	0.93
	**2.78**	1.90	0.39		**2.67**	1.68	0.37
	**3.18**	1.89	0.37		**3.06**	1.68	0.35
	**3.48**	1.90	0.36		**3.47**	1.68	0.37
	**3.48**	1.90	0.38		**3.43**	1.72	0.37

14	**3.30**	1.89	0.49	14	**3.22**	1.67	0.46
	**1.90**	1.90	0.37		**1.96**	1.69	0.33
	**2.70**	1.90	0.37		**2.54**	1.68	0.36
	**3.14**	1.90	0.36		**2.99**	1.68	0.36
	**3.43**	1.89	0.37		**3.13**	1.67	0.41
	**3.77**	1.89	0.38		**3.76**	1.74	0.36
	**4.02**	1.89	0.46		**3.79**	1.68	0.46

17	**2.00**	1.89	0.33	17	**2.28**	1.69	0.37
	**2.66**	1.90	0.35		**2.46**	1.68	0.35
	**3.06**	1.90	0.34		**2.86**	1.69	0.34
	**2.97**	1.89	0.33		**2.81**	1.67	0.32
	**2.86**	1.89	0.52		**2.86**	1.68	0.47
	**3.41**	1.89	0.49		**3.38**	1.67	0.45
	**1.91**	1.89	0.28		**2.87**	1.72	1.13

21	**2.55**	1.96	0.32	21	**2.40**	1.73	0.39
	**3.10**	1.90	0.34		**3.27**	1.68	0.58
	**3.28**	1.90	0.32		**3.45**	1.68	0.37
	**2.87**	1.90	0.46		**3.04**	1.68	0.74
	**2.25**	1.94	0.87		**2.51**	1.70	0.86
	**2.14**	1.92	0.33		**2.40**	1.80	0.37
	**2.73**	2.01	0.38		**2.48**	1.77	0.39
	**3.06**	1.94	0.33		**2.77**	1.73	0.37

25	**3.12**	1.94	0.34	25	**2.99**	1.74	0.34
	**2.65**	1.94	0.75		**2.59**	1.72	0.68
	**3.15**	1.93	0.55		**3.09**	1.70	0.52
	**2.21**	1.92	0.48		**2.83**	1.70	0.84
	**2.68**	1.93	0.36		**2.69**	1.70	0.54
	**3.07**	20.6	0.33		**3.42**	1.85	0.53
	**2.32**	1.94	0.55		**2.37**	1.72	0.55
	**2.97**	1.99	0.69		**3.07**	1.76	0.63

Mean	**2.86**	1.91	0.42	Mean	**2.90**	1.71	0.48
SD	**0.52**	0.03	0.13	SD	**0.42**	0.06	0.18
SEM	**0.08**	0.01	0.02	SEM	**0.06**	0.01	0.03

*The “CBC” RC3 model failed to meet the critical three assumptions of Linear-Least-Squares and was therefore NOT ACCEPTED as statistically sound for estimating H-ARS RC3.

^†^The “CBC-SCHEM” RC3 model met the critical three assumptions of Linear-Least-Squares and was therefore ACCEPTED as statistically sound for estimating H-ARS RC3.
